# Evaluation of *in vitro* effects of various targeted drugs on plasma cells and putative neoplastic stem cells in patients with multiple myeloma

**DOI:** 10.18632/oncotarget.11593

**Published:** 2016-08-25

**Authors:** Katharina Blatt, Harald Herrmann, Gabriele Stefanzl, Wolfgang R. Sperr, Peter Valent

**Affiliations:** ^1^ Ludwig Boltzmann Cluster Oncology, Medical University of Vienna, Vienna, Austria; ^2^ Department of Internal Medicine I, Division of Hematology & Hemostaseology, Medical University of Vienna, Vienna, Austria; ^3^ Department of Radiation Oncology, Medical University of Vienna, Vienna, Austria

**Keywords:** myeloma, molecular targets, targeted drugs, neoplastic stem cells

## Abstract

Multiple myeloma (MM) is a malignancy characterized by monoclonal paraproteinemia and tissue plasmocytosis. In advanced MM cytopenia and osteopathy may occur. Although several effective treatment strategies have been developed in recent years, there is still a need to identify new drug targets and to develop more effective therapies for patients with advanced MM. We examined the effects of 15 targeted drugs on growth and survival of primary MM cells and 5 MM cell lines (MM.1S, NCI-H929, OPM-2, RPMI-8226, U-266). The PI3-kinase blocker BEZ235, the pan-BCL-2 inhibitor obatoclax, the Hsp90-targeting drug 17AAG, and the Polo-like kinase-1 inhibitor BI2536, were found to exert major growth-inhibitory effects in all 5 MM cell lines tested. Moreover, these drugs suppressed the *in vitro* proliferation of primary bone marrow-derived MM cells and induced apoptosis at pharmacologic drug concentrations. Apoptosis-inducing effects were not only seen in the bulk of MM cells but also in MM stem cell-containing CD138^−^/CD20^+^/CD27^+^ memory B-cell fractions. Synergistic growth-inhibitory effects were observed in MM cell lines using various drug combinations, including 17AAG+BI2536 in MM.1S, OPM-2, RPMI-8226, and U-266 cells, 17AAG+BEZ235 in MM.1S, OPM-2, RPMI-8226, and U-266 cells, 17AAG+obatoclax in MM.1S, NCI-H929, OPM-2, and RPMI-8226 cells, BI2536+BEZ235 in MM.1S, NCI-H929, OPM-2, and RPMI-8226 cells, BI2536+obatoclax in MM.1S, OPM-2 and RPMI-8226 cells, and BEZ235+obatoclax in MM.1S and RPMI-8226 cells. Together, our data show that various targeted drugs induce profound and often synergistic anti-neoplastic effects in MM cells which may have clinical implications and may contribute to the development of novel treatment strategies in advanced MM.

## INTRODUCTION

Multiple myeloma (MM) is a hematopoietic neoplasm characterized by an expansion of clonal plasma cells (PC) in the bone marrow (BM) and by an excessive production of monoclonal immunoglobulins (Ig), usually of the IgG- or IgA type [1–3]. Growth, survival, and differentiation of MM cells are triggered by various pro-oncogenic signalling pathways as well as by cytokines and the BM microenvironment [4–9]. Depending on their growth-rate and biologic behaviour, MM cells proliferate and expand in the BM and other organs and subsequently cause organ damage [1–5]. As a result, patients with advanced MM are suffering from pancytopenia, osteolyses, and/or kidney damage. In addition, MM patients often suffer from diffuse osteoporosis and secondary immunoglobulin deficiency [1–4, 10, 11]. During the past 2 decades, several different molecular and cytogenetic risk factors predicting the clinical course and progression in MM have been identified [12–19]. In a considerable number of patients, MM develops from a premalignant condition referred to as monoclonal gammopathy of undetermined significance (MGUS) [20].

Treatment of progressive MM is usually based on cytoreductive agents, glucocorticosteroids, and novel targeted drugs. In the last few years, several attempts have been made to improve treatment of MM by combining novel targeted drugs with each other or with conventional chemotherapy [21–25]. However, despite impressive results, a relatively high percentage of patients develop drug resistances over time. For high risk patients and relapsed MM, more intensive treatments are available, including poly-chemotherapy and autologous or allogeneic stem cell transplantation [21, 26–28]. However, in most cases, MM is still an incurable malignancy. Therefore, there is still a need to identify novel drug-targets and more effective targeted drugs and drug combinations for patients with advanced MM.

Recently, a number of signalling pathways and molecules critically involved in the regulation of growth and survival of MM cells have been identified. These include, among others, the Notch-signalling pathway, the Hedgehog pathway, the phosphoinositide 3-kinase (PI3K), the mammalian target of rapamycin (mTOR) pathway, and the ubiquitin-proteasome pathway [29–36]. Furthermore, the heat shock protein 90 (HSP90) inhibitor 17AAG and the histone deacetylase (HDAC) inhibitor vorinostat were found to exert substantial anti-neoplastic effects on MM cells [37–39]. Other potential drug targets in MM cells are members of the B-cell lymphoma (BCL)-2 family and Polo-like kinase 1 (PLK-1) [40–42].

The concept of neoplastic stem cells has been introduced some time ago, with the intention to explain cellular hierarchies in malignant clones and to define critical target cell populations that exhibit long-term disease-propagating capacity [43–46]. In many neoplasms, these cells are extremely immature and represent a minority in the clone. Based on their selective potential to propagate the malignancy for unlimited time periods, these cells represent a most critical target cell population that needs to be attacked and eliminated in curative treatment approaches [47–49]. In myeloid neoplasms, such as acute myeloid leukemia (AML), the disease-initiating stem- and progenitor cells are considered to reside within a CD34^+^ compartment of the clone [43–46]. By contrast, in MM, the phenotype of neoplastic stem cells (MM stem cells = MMSC) is a matter of debate [52–60]. In several studies, the mouse-repopulating MMSC were found to reside within a CD138^−^/CD20^+^/CD27^+^ (or a CD19^+^/CD27^+^/CD138^−^) cell population [52–56]. In other studies, MMSC were found to reside within a CD19^−^/CD45^low^/CD38^+^/CD138^+^ fraction of the clone [56, 57]. In each case, only a small sub-fraction of these cell populations may fulfil true stem cell function. The aims of this study were to examine the effects of various targeted drugs on growth and survival of MM cells, to ask whether novel targeted drugs exert synergistic growth-inhibitory effect, and to explore whether MMSC also respond to these drugs.

## RESULTS

### Various targeted drugs inhibit proliferation of MM cell lines

As assessed by ^3^H-thymidine uptake, a number of antineoplastic drugs were found to inhibit growth of MM cells at pharmacologically relevant concentrations (Table 1). Four drugs blocked proliferation in all five MM cell lines tested: the PLK-1 inhibitor BI2536 (IC_50_ 0.01-0.05 μM), the pan-BCL-2 antagonist obatoclax (IC_50_ 0.1-0.5 μM), the HSP90 inhibitor 17AAG (IC_50_ 0.01-1 μM), and the PI3 kinase/mTOR inhibitor BEZ235 (IC_50_ 0.01-1 μM) (Table 1). The Aurora-kinase-targeting drug VX-680 inhibited growth of MM.1S, NCI-H929, and OPM-2 cells (IC_50_ 0.1-1 μM). The HDAC-inhibitor vorinostat suppressed the proliferation of MM.1S, NCI-H929, and U-266 cells (IC_50_ 0.1-1 μM). In the other MM cell lines, vorinostat showed no effects on proliferation below 1 μM. Sunitinib blocked proliferation of MM.1S cells and U-266 cells (IC_50_ 0.1 −1 μM) but did not inhibit growth in the other MM cell lines (Table 1). Similarly, nilotinib suppressed growth of MM.1S and U-266 cells (IC_50_ 0.5-1 μM) but not inhibit growth in the other MM cell lines (IC_50_ >1 μM). A summary of the drug effects on growth of MM cell lines is shown in Table 1.

**Table 1:** Effects of various targeted drugs on proliferation of myeloma cell lines

Drug	IC_50_ values (μM) produced by various drugs in				
	MM.1S	NCI-H929	OPM-2	RPMI-8226	U-266
BI2536	0.01	0.01-0.05	0.01-0.05	0.01-0.05	0.01-0.05
Obatoclax	0.1-0.5	0.5	0.1-0.5	0.5	0.5
BEZ235	0.1-0.5	<0.01	0.1-0.5	1	0.1-0.5
17AAG	0.01-0.05	0.01-0.05	0.5-1	0.5-1	0.1-0.5
VX-680	0.1-0.5	0.5-1	0.5-1	>10	1-5
Vorinostat	0.5	0.5-1	>1	1	0.1-0.5
Nilotinib	0.5-1	>1	>1	>1	0.5-1
Imatinib	10	>10	>10	>10	>10
Dasatinib	5-10	>10	>10	>10	5-10
Bosutinib	5-10	5	1-5	5-10	1-5
Sorafenib	1-5	1-5	1-5	1-5	1-5
Sunitinib	0.1-0.5	1-5	1-5	5-10	0.5-1
Erlotinib	1-5	>10	>10	>10	5-10
Gefitinib	1-5	>10	>10	>10	5-10
Lapatinib	1-5	10	5-10	>10	5-10

MM cell lines were incubated with increasing concentrations of various drugs (0.001-10 μM) at 37°C for 48 hours. After incubation, 0.5 μCi of ^3^H-thymidine was added to each well and kept for 16 hours (37°C). Proliferation was measured by analyzing ^3^H-thymidine uptake. Results are expressed as inhibitory concentration producing 50% inhibition (IC_50_).

### Effects of targeted drugs on *in vitro* proliferation of primary MM cells

**Figure 1:**
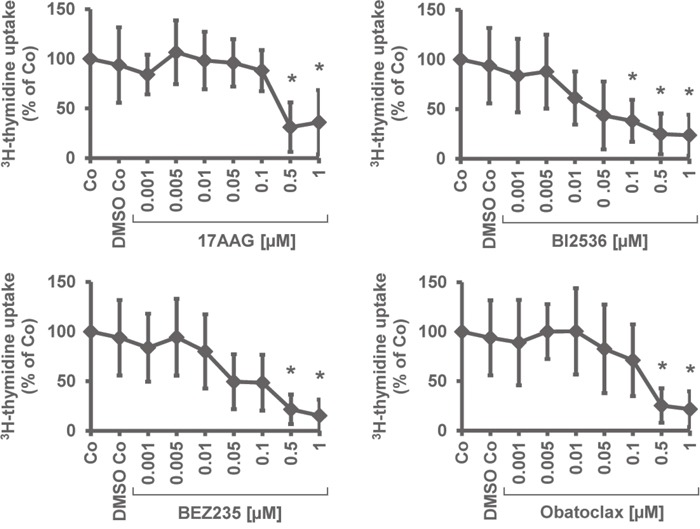
Effects of 17AAG, BI2536, obatoclax, and BEZ235 on proliferation of primary neoplastic MM cells Primary BM cells obtained from 6 patients with MM were incubated in control medium (Co), DMSO control, or in various concentrations of 17AAG, BI2536, BEZ235, or obatoclax (0.001-1 μM) at 37°C for 48 hours. Thereafter, uptake of ^3^H-thymidine was measured. Results are expressed as percent of medium control and represent the mean±S.D. from 6 independent experiments. Asterisk (*): p<0.05.

In a next step, we examined the effects of 17AAG, BI2536, BEZ235, and obatoclax on *in vitro* proliferation of primary neoplastic PC obtained from the BM of patients with MM. The patients’ characteristics are shown in Table 2. We found that all 4 drugs tested exert dose-dependent growth-inhibitory effects in primary MM cells, with pharmacologically relevant IC_50_ values (Table 3). Figure 1 shows a summary of growth-inhibitory effects obtained with the 4 drugs in the primary cell samples tested. IC_50_ values obtained with primary BM cells (PC) were found to be within a pharmacological range and to correspond to IC_50_ values obtained with the MM cell lines tested (Figure 1, Tables 1 and 3).

**Table 2:** Characteristics of multiple myeloma patients

Pat No	sex [f/m]	age* [years]	PC** [%]	PC*** [%]	Ig type [g/L]	Stage Durie/Salmon	WBC [x10^9^/L]	Hb [g/dL]	PLT [x10^9^/L]	β2-microglobulin [mg/L]	BM cytogenetics	time of sampling
1	m	53	90	42	IgG lambda [83.7]	III	3.44	9.5	148	8.11	46,XY	diagnosis
2	m	84	15	14	lambda [32.9]	II	7.58	11.8	335	21.1	46,XY	diagnosis
3	f	68	95	34	IgA kappa [33.10]	II	3.21	8.3	98	9.52	complex	diagnosis
4	m	48	80	77	IgG kappa [23.4]	III	6.11	9.9	169	3.73	not done	diagnosis
5	m	85	70	11	IgA kappa [24.4]	III	4.73	8.6	294	4.23	46,XY	diagnosis
6	f	69	70	18	IgA lambda [57.2]	I	8.16	9.3	338	9.81	47,XX,+17	diagnosis
7	f	71	80	21-22	IgA kappa [19.3]	II	7.16	10.4	420	5.52	complex	diagnosis
8	f	54	90	>90	IgG kappa [56.3]	I	3.73	8.9	38	5.79	complex	relapse

Pat No, patient number; m, male; f, female; PC, plasma cells; WBC, white blood count; Hb, hemoglobin; PLT, platelets; BM, bone marrow; n.t., not tested. *Age at the time of BM sampling (in most cases BM was obtained at diagnosis). **Percentage of plasma cells (of all nucleated BM cells) in immunohistochemical examinations in BM sections. ***Percentage of plasma cells (of all nucleated BM cells) in BM smears

**Table 3:** Effects of the most effective targeted drugs on proliferation of primary neoplastic BM cells

Pat No	sex	age	PC** [%]	PC*** [%]	IC_50_ (μM) values produced by			
					17AAG	BI2536	BEZ235	obatoclax
1	m	53	90	42	0.1-0.5	0.05	0.01-0.05	0.1-0.5
2	m	84	15	14	0.5	0.005-0.01	0.005-0.01	0.01-0.05
3	f	68	95	34	0.5-1	0.1	0.01-0.05	0.1-0.5
6	f	69	70	18	0.1-0.5	0.001	0.005-0.01	0.01-0.05
7	f	71	80	21-22	0.1-0.5	0.01-0.05	0.1-0.5	0.1-0.5
8	f	54	90	>90	0.5-1	0.5	0.1-0.5	0.5

BM, bone marrow; Pat No, patient number; m, male; f, female; PC, plasma cells. **Percent plasma cells (of all nucleated BM cells) are based on immunohistochemical examinations in BM sections. ***Percentage of plasma cells (of all nucleated BM cells) in BM smears. Primary MM cells were incubated with increasing concentrations of 17AAG, BI2536, obatoclax, or BEZ235 (0.001-1 μM) at 37°C for 48 hours. After incubation, 0.5 μCi of ^3^H-thymidine was added for 16 hours. Proliferation was measured by analyzing ^3^H-thymidine uptake. Abbreviations: IC_50_, inhibitory concentration (50%).

### Various targeted drugs induce apoptosis in MM cell lines

**Figure 2:**
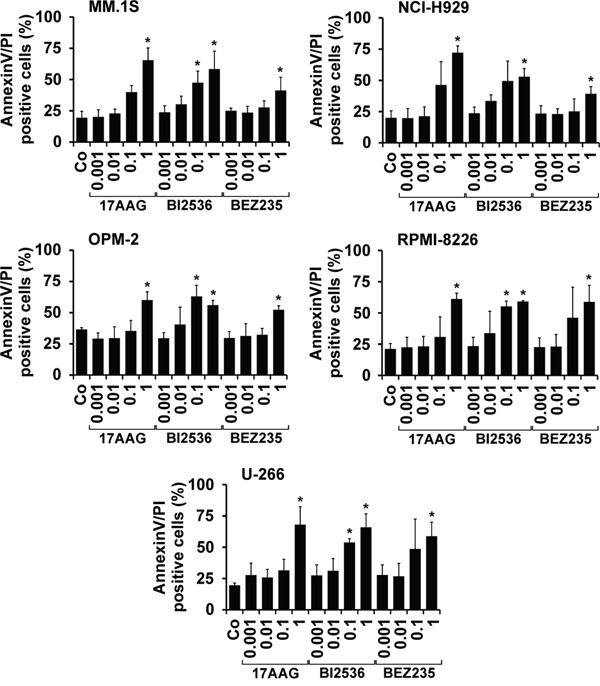
Effects of 17AAG, BI2536, and BEZ235 on growth of MM cell lines MM cell lines (MM.1S, NCI-H929, OPM-2, RPMI-8226, U-266) were incubated in control medium (Co) or in various concentrations of 17AAG, BI2536, or BEZ235 (0.001-1 μM) at 37°C for 48 hours. Then, the percentage of apoptotic cells was determined by AnnexinV/PI staining and flow cytometry. Results show the percentage of AnnexinV/PI+ cells and represent the mean±S.D. from 3 independent experiments. Asterisk (*): p<0.05.

To define the mechanism of drug action, we examined drug effects on survival and apoptosis in MM cells. Apoptosis was quantified by analyzing expression of active caspase-3 by flow cytometry (Table 4). BI2536, obatoclax, BEZ235, and 17AAG produced dose-dependent apoptosis in all 5 MM cell lines tested. The most effective drugs were BI2536 (EC_50_ 0.001-0.01 μM) and obatoclax (EC_50_ 0.001-0.5 μM), followed by BEZ235 (EC_50_ 0.01-0.5 μM) and 17AAG (EC_50_ 0.01-1 μM). The HDAC blocker VX-680 induced growth inhibition in OPM-2, RPMI-8226, and U-266 cells (EC_50_ 0.1-1 μM), whereas the other cell lines tested did not respond to VX-680 (Table 4). Vorinostat induced apoptosis in U-266 cells (EC_50_ 0.5-1 μM) but did not produce apoptosis in the other MM cell lines. Sunitinib was found to exert apoptosis-inducing effects in MM.1S, OPM-2, RPMI-8226, and U-266 cells (EC_50_ 0.5-1 μM) (Table 4). The effects of the most potent drugs (17AAG, BI2536, BEZ235) on survival of MM cells was confirmed by Annexin V/PI staining, with similar EC_50_ values compared to that obtained by staining for active caspase-3 (Figure 2).

**Table 4:** Effects of various targeted drugs on survival (apoptosis) of myeloma cell lines

Drug	EC_50_ (μM) values produced by various drugs in				
	MM.1S	NCI-H929	OPM-2	RPMI-8226	U-266
BI2536	0.001-0.01	0.01	0.001-0.01	0.001-0.01	0.001-0.01
Obatoclax	0.5	0.5	0.001-0.01	0.01-0.1	0.5
BEZ235	0.1-0.5	0.5	0.01-0.1	0.5	0.1-0.5
17AAG	0.01-0.1	0.1-0.5	0.1-0.5	0.5	1
VX-680	5	1-5	0.5-1	0.1-0.5	0.5
Vorinostat	1-5	1-5	1	1-5	0.5-1
Nilotinib	1-5	1-5	1-5	1-5	1-5
Imatinib	>10	>10	>10	>10	>10
Dasatinib	>10	>10	5-10	>10	>10
Bosutinib	1-5	5	1-5	1-5	5
Sorafenib	5	1-5	1-5	5	1-5
Sunitinib	1	1-5	0.5-1	0.5	1
Erlotinib	>10	>10	>10	>10	>10
Gefitinib	>10	>10	>10	>10	>10
Lapatinib	5-10	>10	5-10	5-10	5-10

Myeloma cell lines were incubated with increasing concentrations of various drugs (0.001-10 μM) at 37°C for 48 hours. Apoptosis was determined by active caspase-3 staining and flow cytometry as described in the text. Abbreviations: EC_50_, effective concentration (50%).

### Drug effects on survival (apoptosis) of primary MM cells, putative MM stem cells (MMSC), CD34^+^/CD38^−^ cells, and CD34^+^/CD38^+^ cells

**Figure 3:**
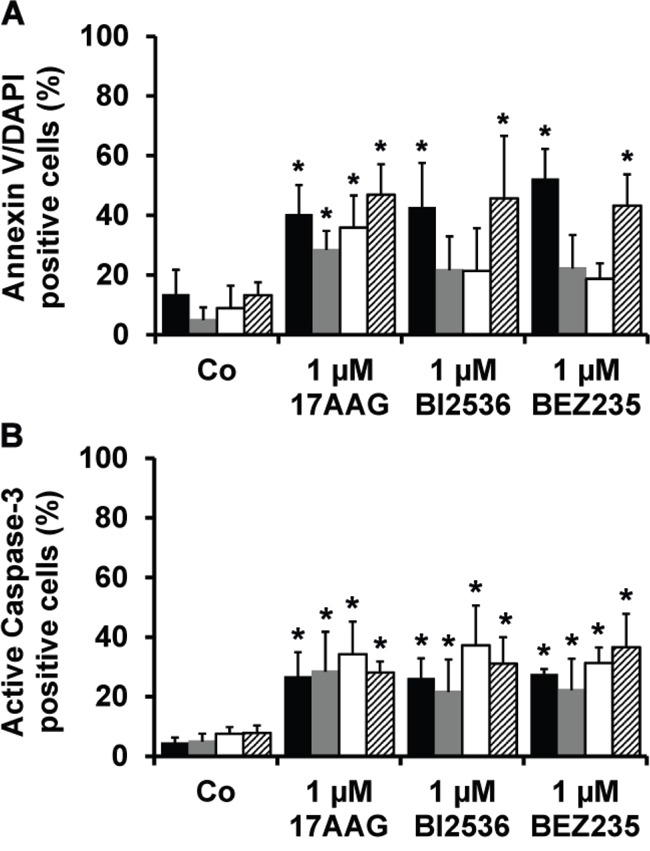
Effects of 17AAG, BI2536, and BEZ235 on survival of primary MM cells Primary BM cells derived from 6 patients with MM were incubated in control medium (Co) or in medium containing 17AAG, BI2536, or BEZ235 (each 1 μM) at 37°C for 48 hours. Thereafter, cells were stained with antibodies against AnnexinV **A.** or active caspase-3 **B.** by multicolor flow cytometry as described in the text. The following subsets of cells were examined: CD138^+^ MM cells (black bars), CD138^−^/CD27^+^/CD20^+^ putative MMSC (grey bars), CD34^+^/CD38^−^ hematopoietic stem cells (open bars), and CD34^+^/CD38^+^ cells (hatched bars). Results are expressed as percent AnnexinV+ cells (A) or percent active caspase-3+ cells (B) and represent the mean±S.D. from 6 independent experiments. Asterisk (*): p<0.05.

In a next step, the most potent drugs (17AAG, BI2536, BEZ235) were examined for their effects on survival of primary BM-derived plasma cells, putative neoplastic MM stem cells, CD34^+^/CD38^−^ hematopoietic stem cells (HSC) and CD34^+^/CD38^+^ hematopoietic progenitor cells by staining for active caspase-3 and Annexin V/DAPI. As shown in Figure 3, all three drugs tested (17AAG, BI2536, BEZ235) induced apoptosis in CD138^+^ MM cells as well as in CD138^−^/CD20^+^/CD27^+^ MMSC-containing cell fractions in all donors tested (Figure 3). However, we also found that all three drugs produce apoptosis in presumably normal CD34^+^/CD38^−^ HSC and CD34^+^/CD38^+^ progenitor cells (Figure 3).

### 17-AAG, BI2536, and BEZ235 inhibit cell cycle progression in MM cells

**Figure 4:**
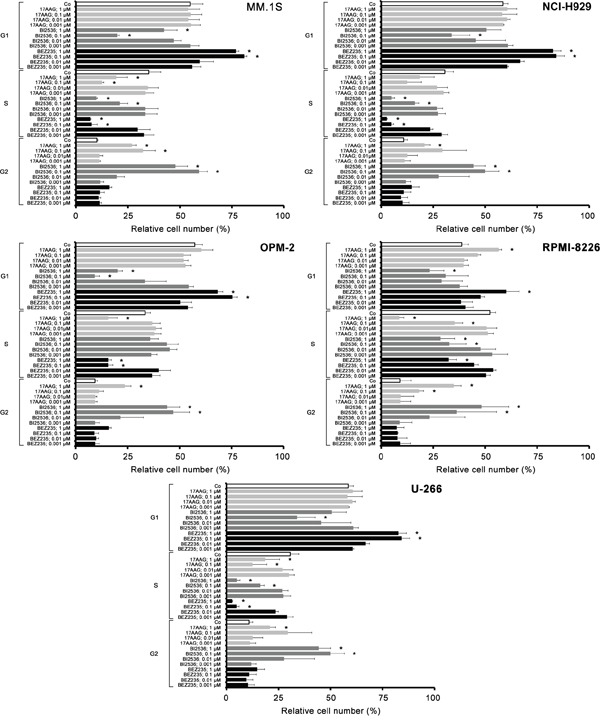
Effects of 17AAG, BI2536, and BEZ235 on cell cycle progression in MM cell lines MM.1S cells (upper left panel), NCI-H929 cells (upper right panel), OPM-2 cells (middle left panel), RPMI-8226 cells (middle right panel) and U-266 cells (lower panel) were incubated in control medium (Co) or various concentrations of 17AAG, BI2536, or BEZ235 (0.001-1 μM each) at 37°C for 48 hours. Then, cell cycle distribution was analyzed by flow cytometry as described in the text. Asterisk (*): p<0.05.

We next examined the effects of 17AAG, BI2536, and BEZ235 on cell cycle progression in the 5 MM cell lines tested. As expected, the PI3 kinase/mTOR blocker BEZ235 produced a G1 cell cycle arrest in all MM cell lines tested, whereas 17AAG and BI2536 induced a G2 cell cycle arrest in these cells (Figure 4). The effects of 17AAG and BI2536 on cell cycle progression were weaker compared to the effects produced by BEZ235 (Figure 4).

### Identification of drug combinations producing synergistic growth-inhibitory effects

**Figure 5:**
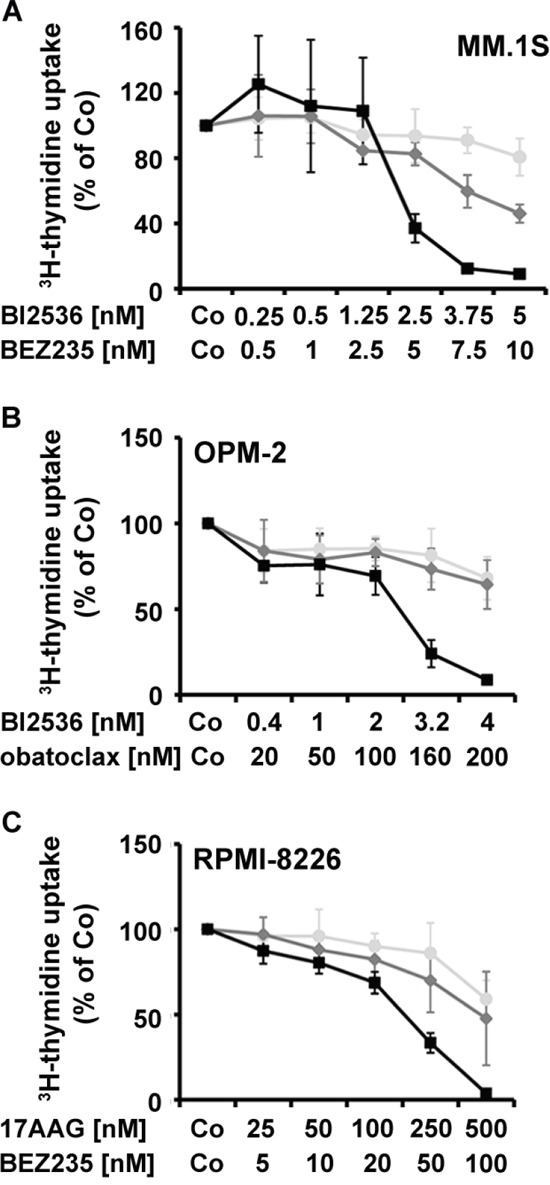
Effects of drug combinations on proliferation in MM cells MM.1S cells **A.**, OPM-2 cells **B.**, and RPMI-8226 cells **C.** were incubated in control medium (Co), in medium containing individual drugs alone, or in medium containing drug combinations (at fixed ratio) at 37°C for 48 hours. Then, uptake of ^3^H-thymidine was measured. MM.1S cells (A) were incubated in various concentrations of BI2536 (○-○), BEZ235 (◇-◇), or combinations of both drugs (◾-◾). OPM-2 cells (B) were incubated with various concentrations of BI2536 (○-○) or obatoclax (◇-◇) or combinations of both drugs (○-○). RPMI-8226 cells (C) were incubated with various concentrations of 17AAG (○-○) or BEZ235 (◇-◇) or combinations of both drugs (○-○). Results show the percentage of ^3^H-thymidine uptake compared to medium control and represent the mean±S.D. of one typical experiment.

Based on the encouraging results obtained with several of the targeted drugs applied and because of their potential toxicity, we screened for cooperative anti-neoplastic drug effects in the 5 MM cell lines employed. Drug combinations were classified as additive, antagonistic, or synergistic by CalcuSyn software. A summary of drug-combination effects is shown in Table 5. Clear synergistic growth-inhibitory effects were observed when applying the drug combinations 17AAG+BI2536 and 17AAG+BEZ235 in MM.1S, OPM-2, RPMI-8226, and U-266 cells, 17AAG+obatoclax and BI2536+BEZ235 in MM.1S, NCI-H929, OPM-2, and RPMI-8226 cells, BI2536+obatoclax in MM.1S, OPM-2, and RPMI-8226 cells, and BEZ235+obatoclax in MM.1S and RPMI-8226 cells (Table 5). Figure 5 shows examples of synergistic drug interactions obtained in drug combination experiments in MM cell lines.

**Table 5:** Effects of various drug combinations on proliferation of myeloma cell lines

Drug combinations	Drug interactions produced in				
	MM.1S	NCI-H929	OPM-2	RPMI-8226	U-266
17AAG and BI2536	s	an	s	s	s
17AAG and BEZ235	s	an	s	s	s
17AAG and obatoclax	s	s	s	s	an
BI2536 and BEZ235	s	s	s	s	an
BI2536 and obatoclax	s	an	s	s	an
BEZ235 and obatoclax	s	ad	an	s	an

To determine potential additive or synergistic drug effects, MM cell lines were exposed to 17AAG, BI2536, BEZ235, and obatoclax, alone or in combination at a fixed ratio of drug concentrations at 37°C for 48 hours. Proliferation was measured by analyzing ^3^H-thymidine uptake. Drug interactions (synergistic = s, additive = ad, and antagonistic = an) were calculated by CalcuSyn software. A combination index (CI) of <1 indicates synergism, CI values of 1 indicate additive effects, and CI values >1 indicate antagonistic drug effects.

### Identification of molecular targets expressed in MM cell lines

As assessed by qPCR, all MM cell lines expressed transcripts specific for PLK-1, PI3K, mTOR, BCL-2, and the myeloid cell leukemia 1 (MCL-1) antigen (Table 6). BCL-xL mRNA was detected in MM.1S, RPMI-8226, and U-266 cells, but not in NCI-H929 and OPM-2 cells. No definitive correlation between expression of molecular targets (or target-patterns) and responses to individual drugs or drug combinations was found.

**Table 6:** Molecular targets expressed in myeloma cell lines

Drug targets	% of human ABL mRNA				
	MM.1S	NCI-H929	OPM-2	RPMI-8226	U-266
PLK-1	++	++	++	++	++
PI3K	++	++	+	++	++
mTOR	++	++	++	++	++
BCL-2	++	++	++	++	+
BCL-xL	+/−	-	-	+	++
MCL-1	++	++	++	++	++

To quantify mRNA expression levels in myeloma cell lines, qPCR was performed using primers specific for PLK-1, PI3K, mTOR, BCL-2, BCL-xL, MCL-1, and ABL (Table 7). mRNA levels were expressed as percentage of ABL. The following score of mRNA expression was applied (% of ABL): <10%: −; 10%-29%: +/−; 30%-100%:+; >100%: ++.

PLK-1: polo-like kinase 1; PI3K: phosphoinositide 3-kinase; mTOR: mammalian target of rapamycin; BCL-2: B-cell lymphoma 2; BCL-xL: B-cell lymphoma-extra large; MCL-1: myeloid cell leukemia 1.

## DISCUSSION

During the past few years, therapy of MM improved considerably and resulted in better progression-free and overall survival. However, still, not all patients with MM enter long-term progression-free survival after therapy. Overall, there is a need to develop new more effective targeted drugs and drug combinations for these patients. In the past few years, several novel targeted drugs have shown promising results in preclinical studies [21–25, 29, 31–33, 36–42]. In the current study, we have extended these analyses by examining the effects of various targeted drugs on growth and survival of primary MM cells and putative MMSC, and by combining most effective targeted drugs with each other. Of the 15 drugs tested in this study, 17AAG, BI2536, BEZ235, and obatoclax were identified as potent inhibitors of growth and survival of MM cells. In addition, we found that all 4 drugs block growth and survival of primary MM cells, and that drug combinations elicit additive or even synergistic growth-inhibitory effects. These data may have clinical implications and may pave the way for the development of novel more effective anti-MM therapies.

A number of different signaling pathways and survival molecules contribute to growth and survival of MM cells [29–36]. During the past 15 years, targeted drugs have been developed that interfere with these pathways and block specific signaling molecules or survival-related molecules in neoplastic cells [32, 33, 37–42]. In our study, 15 targeted drugs were applied, and 4 of these drugs were found to act as potent inhibitors of growth and survival of MM cells. These drugs are the Hsp90 targeting drug 17AAG, the PLK-1 inhibitor BI2536, the dual PI3 kinase/mTOR blocker BEZ235, and the pan BCL-2 inhibitor obatoclax. The IC_50_ values produced by these drugs were found to be in a pharmacologically relevant range. In addition, these drugs suppressed cell cycle progression and induced apoptosis in MM cells. We were also able to show that the molecular targets through which these drugs exert their growth-inhibitory effects, namely PLK-1, PI3K, mTOR, BCL-2, BCL-xL, and MCL-1, are expressed in MM cells. Whereas most targets were expressed abundantly in MM cell lines, BCL-xL mRNA was only expressed at low levels or was not detected in the MM cell lines tested. We also tried to correlate (synergistic) drug effects with target expression patterns in MM cells. However, no clear correlation between expression of molecular targets and responses to individual drugs or drug combinations was found. This is best explained by the fact that several different (known and unknown) targets are recognized by these drugs and contribute to the observed drug effects.

Cell line models are a useful tool for screening potential drug effects in various cancer entities. However, primary cells may behave differentially and often show different responses to targeted drugs. Therefore, we examined drug effects on primary MM cells. We found that 17AAG, BI2536, BEZ235, and obatoclax induce growth inhibition in primary BM-derived MM cells in all patients tested. The IC_50_ values were comparable among patients, independent of the percentage of neoplastic cells in BM samples, type of MM, or disease status (diagnostic sample versus relapse). Although all 4 drugs exerted potent effects, slightly lower IC_50_ values were obtained with BI2536 and BEZ235 compared to 17AAG and obatoclax. It is also noteworthy that the IC_50_ values obtained with primary MM cells corresponded to IC_50_ values obtained in MM cell lines.

In the past 15 years, neoplastic stem cells have been identified as a novel relevant target of therapy in malignant blood cell disorders [47–49]. Any type of therapy can only be curative when eliminating these disease-propagating cells. In MM, the phenotype of neoplastic stem cells (MMSC) is still a matter of debate [45, 52–60]. In the past few years evidence has accumulated to suggest that these cells may reside as rare subset in a CD138^−^/CD20^+^/CD27^+^ population of the clone [52–56]. In the present study, we examined the effects of the 4 most potent drugs on survival of these putative MMSC. We found that these drugs induce apoptosis not only in the bulk of MM cells but also in putative MMSC, which may have clinical implications. However, these studies were performed *in vitro*, and additional studies using these drugs in suitable xenotransplantation assays performed with primary MM cells would be required to confirm their MMSC-eliminating activity. Unfortunately, however, no robust xenotransplantation model is available in MM. Once such model is available, we will examine drug effects on MMSC engraftment in future studies.

Most of the targeted drugs examined may also exert growth-inhibitory effects on normal blood and BM cells. In the current study, we examined the effects of the 4 most potent targeted drugs on survival of CD34^+^/CD38^−^ HSC and CD34^+^/CD38^+^ progenitor cells. In these experiments all 4 drugs induced apoptosis in normal BM stem and progenitor cells at 1 μM. These data suggest that most of these drugs can produce cytopenia in patients which corresponds with clinical observations. An alternative possibility may be that some of the CD34^+^ cells were immature clonal cells. However, although this hypothesis has been propagated by some investigators [61, 62] others have concluded that clonal MM progenitors do not reside within CD34^+^ cells [63].

Based on the obvious risk of side effects and the observation that synergistic drug effects can often be obtained, anti-MM therapy usually consists of various drug combinations. In the present study, we were interested to learn whether the most effective targeted drugs identified would also show cooperative or even synergistic anti-proliferative effects on MM cells when applied in combination. Indeed, we found that various combinations of the targeted drugs applied produce clear synergistic effects on growth of MM cell lines. The most potent synergistic effects were seen when combining BI2536 and BEZ235 in MM.1S or BI2536 and obatoclax in OPM-2 and 17AAG and BEZ235 in RPMI-8226 cells. Remarkably, in MM.1S cells and RPMI-8226 cells, all drug combinations applied showed synergistic effects. These data suggest that it may be reasonable to apply such drug combinations also *in vivo* once the individual drugs have shown to act anti-neoplastic in patients. By employing such combination strategies, drug-induced toxicity may also be reduced.

In conclusion, our data show that various targeted drugs exert major growth-inhibitory and apoptosis-inducing effects on primary MM cells, their putative stem cells, and MM cell lines, and that these effects can be further augmented by applying drug combinations. Clinical trials are now warranted in order to confirm these effects *in vivo* in patients with MM. The most obvious clinical need may be patients with relapsed or refractory MM [64, 65].

## MATERIALS AND METHODS

### Reagents

A number of anti-neoplastic drugs were tested for their ability to inhibit growth of MM cells: the tyrosine kinase inhibitors (TKI) bosutinib, dasatinib, imatinib, sorafenib, sunitinib, and nilotinib, the ErbB-receptor inhibitors lapatinib, erlotinib, and gefitinib, the Aurora-kinase inhibitor VX-680, the HSP90 inhibitor 17AAG, the PLK-1 inhibitor BI2536, the pan-BCL-2 antagonist obatoclax, and the HDAC-inhibitor vorinostat were purchased from Chemietek (Indianapolis, IN, USA). The PI3 kinase/mTOR inhibitor BEZ235 was obtained from Selleck Chemicals (Houston, TX, USA). Stock solutions of drugs were prepared by dissolving in dimethylsulfoxide, DMSO (Merck, Darmstadt, Germany). RPMI 1640 medium and fetal calf serum (FCS) were purchased from PAA Laboratories (Pasching, Austria), and ^3^H-thymidine from PerkinElmer (Waltham, MA, USA). FITC-labeled CD34 monoclonal antibody (mAb) 581, PE-labeled CD34 mAb 581, FITC-labeled CD138 mAb MI15, PE-labeled CD138 mAb DL-101, PerCP-labeled CD45 mAb 2D1, APC-labeled CD38 mAb HIT2, PE-labeled and Alexa Fluor® 647-labeled active caspase-3 mAb C92-605 were purchased from BD Biosciences (San Jose, CA, USA). The PerCP-labeled CD20 mAb 2H7 and the APC-labeled CD27 mAb O323 were obtained from Biolegend (San Diego, CA, USA), and an Annexin V/FITC kit from eBioscience (San Diego, CA, USA).

### Culture of MM cells

The MM cell lines NCI-H929, OPM-2, RPMI-8226 and U-266 were obtained from the German Collection of Microorganisms and Cell Cultures (DMSZ; Braunschweig, Germany) and MM.1S from American Type Culture Collection (ATCC; Manassas, VA, USA). Cell lines were cultured in RPMI1640 with 10% FCS and antibiotics at 5% CO_2_ and 37°C. Cells were passaged every 2-3 days and re-thawed from an original stock every 6-8 weeks. The biologic stability of these cell lines was checked by cell surface phenotyping (flow cytometry). Primary BM cells were obtained (routine investigations) from 8 patients with MM after written informed consent was given. Samples were collected at diagnosis, or relapse (Table 2). The study was approved by the ethics committee of the Medical University of Vienna. Primary BM cells were either analyzed by multicolor flow cytometry or were fractionated using Ficoll, in order to obtain mononuclear cells (MNC).

### Flow cytometry and characterization of MMSC

Heparinized BM cells (10^6^/tube) were incubated with combinations of mAb for 15 minutes. PC were defined as CD138^+^ cells and the MMSC-containing cell fractions were defined as CD138^−^/CD20^+^/CD27^+^ cells using FITC-labeled CD138 mAb, APC-labeled CD27 mAb, and PerCP-labeled CD20 mAb. Hematopoietic stem cells (HSC) were defined as CD34^+^/CD38^−^ cells and hematopoietic progenitors as CD34^+^/CD38^+^ cells, using FITC-labeled CD34 mAb and APC-labeled CD38 mAb. After erythrocyte-lysis using FACS-Lysing-Solution (BD Biosciences) PC, MMSC, HSC, and hematopoietic progenitor cells were analyzed by multicolor flow cytometry on a FACSCalibur (BD Biosciences) using FlowJo software (TreeStar, Ashland, OR, USA). To study drug effects on survival (apoptosis) of primary PC, MMSC, HSC, and progenitors cells, primary BM MNC of 6 MM patients were incubated in control medium, 17AAG, BI2536, or BEZ235 (1 μM each) at 37°C for 48 hours. Thereafter, cells were washed and examined for apoptosis by combined staining for surface antigens (to define cell populations) and either AnnexinV-FITC plus 4′, 6-diamidino-2-phenylindole (DAPI; Invitrogen, Carlsbad, CA) or with an antibody against active caspase-3 as described [66]. Apoptosis was quantified by measuring the percentage of AnnexinV+ cells and active caspase-3+ cells in various gated cell fractions, namely CD138^+^ PC, CD138^−^/CD20^+^/CD27^+^ MMSC, CD34^+^/CD38^−^ HSC, and CD34^+^/CD38^+^ hematopoietic progenitor cells.

### Evaluation of drug effects on proliferation of MM cell lines and primary MM cells

To study the effects of various drugs on proliferation of MM cell lines, ^3^H-thymidine incorporation experiments were performed. For this purpose, MM.1S, NCI-H929, OPM-2, RPMI-8226 and U-266 cells (10^4^/well) were seeded in 96-well plates (TPP, Trasadingen, Switzerland) and incubated with increasing drug concentrations (0.001-10 μM) at 37°C for 48 hours. In a different set of experiment, primary BM derived cells obtained from 6 patients with MM were incubated in control medium or various concentrations of 17AAG, BI2536, obatoclax, and BEZ235 (0.001-1 μM) at 37°C for 48 hours. After incubation, 0.5 μCi of ^3^H-thymidine was added to each well and kept for 16 hours (37°C). Cells were then harvested on filter membranes (Perkin Elmer, Waltham, MA, USA) in a FilterMate Harvester (Perkin Elmer). Filters were air-dried and the bound radioactivity counted in a ß-counter (MicroBeta^2^ 2450 Microplate Counter, Perkin Elmer). To determine potential additive or synergistic drug effects, MM cells were exposed to various combinations of 17AAG, BI2536, BEZ235, and obatoclax at a fixed ratio of drug concentrations [67]. All experiments were performed in triplicates.

### Evaluation of drug-induced apoptosis in MM cell lines

To assess the effects of targeted drugs on expression of activated caspase-3, flow cytometry experiments were performed using MM cell lines and an antibody against active caspase-3 (C92-605; *BD Biosciences*). In these experiments, cells were cultured in the presence of control medium or targeted drugs at various concentrations (0.001-10 μM) at 37°C for 48 hours. Prior to staining, cells were fixed in formaldehyde (2%), and permeabilized using methanol (100%) at −20°C for 30 minutes. Expression of active caspase-3 was analyzed on a FACSCalibur (BD Biosciences) as decribed [66]. To confirm apoptosis in cell lines after drug exposure, Annexin V/PI staining was performed with the three most effective drugs 17AAG, BI2536, BEZ235 in all five MM cell lines as reported [67]. For cell cycle studies with the three potent drugs 17AAG, BI2536 and BEZ235, drug-exposed cells were resuspended in 500 μL permeabilization buffer (0.1% Na-acetate and 0.1% Triton X-100). Then 40 μl PI were added, and cell cycle distribution analyzed on a FACSCalibur. For obatoclax, the AnnexinV/PI staining and cell cycle analyis was not possible due to the autofluoresence of the drug.

### Quantitative PCR (qPCR)

Total RNA was isolated from the five MM cell lines (MM1.S, NCI-H929, OPM-2, RPMI-8226, U-266) using RNeasy MinElute™ Cleanup Kit (Qiagen, Hilden, Germany). cDNA was synthesized using Moloney murine leukemia virus reverse transcriptase (Invitrogen, Carlsbad, CA, USA), random primers, First-Strand-Buffer, dNTPs, and RNasin (all from Invitrogen) according to the manufacturer's instructions. qPCR was performed as described [66] using iTaq SYBR-Green-Supermix with ROX (Bio-Rad, Hercules, CA) and primers specific for *BCL-2, BCL-xL, MCL-1, PI3K, mTOR* and *PLK-1* (Table 7). BCL-2, BCL-xL, MCL-1, PI3K, mTOR, and PLK-1 mRNA levels were expressed as percentage of ABL transcript levels [66].

**Table 7:** Oligonucleotide sequences of primers used for quantitative PCR (qPCR)

Gene	Sequence
hu PLK-1-fwd	5′-CCC ATC TTC TGG GTC AGC AAG-3′
hu PLK-1-rev	5′-AAG AGC ACC CCC ACG CTG TT-3′
hu PI3K-fwd	5′-TAG CTA TTC CCA CGC AGG AC-3′
hu PI3K-rev	5′-TTG CTT TGA GCT GTT CTT TGT C-3′
hu mTOR-fwd	5′-CCC ACG TTC CTT AAC GTC AT-3′
hu mTOR-rev	5′-GGC TCT TCA CAA AGG ACA CC-3′
hu BCL-2-fwd	5′-TTG ACA GAG GAT CAT GCT GTA CTT-3′
hu BCL-2-rev	5′-TCA GTC TAC TTC CTC TGT GAT GTT GT-3′
hu BCL-xL-fwd	5′-CTC CTC TCC CGA CCT GTG AT-3′
hu BCL-xL-rev	5′-AAG ATT CTG AAG GGA GAG AAA GAG A-3′
hu MCL-1-fwd	5′-GTG CAG CGC AAC CAC GAG-3′
hu MCL-1-rev	5′-CGA TTT CAC ATC GTC TTC GTT T-3′
hu ABL-fwd	5′-TGT ATG ATT TTG TGG CCA GTG GAG-3′
hu ABL-rev	5′-GCC TAA GAC CCG GAG CTT TTC A-3′

hu: human; fwd: forward; rev, reverse; PLK-1: polo-like kinase 1; PI3K: phosphoinositide 3-kinase; mTOR: mammalian target of rapamycin; BCL-2: B-cell lymphoma 2; BCL-xL: B-cell lymphoma-extra large; MCL-1: myeloid cell leukemia 1; ABL: Abelson murine leukemia viral oncogene homolog.

### Statistical evaluation of data

To determine the significance in differences in growth and apoptosis, the Student's t test for dependent samples was applied. Results were considered significant when p<0.05. Drug interactions (additive, synergistic, and antagonistic) were determined by calculating combination index (CI) values using CalcuSyn software (Biosoft, Cambridge, U.K.) [68]. A CI value of 1 indicates an additive effect, whereas CI values below 1 indicate synergistic drug effects, and CI values >1 antagonistic effects.
